# The Interaction between the First Transmembrane Domain and the Thumb of ASIC1a Is Critical for Its *N-*Glycosylation and Trafficking

**DOI:** 10.1371/journal.pone.0026909

**Published:** 2011-10-28

**Authors:** Lan Jing, Yu-Qing Jiang, Qian Jiang, Bin Wang, Xiang-Ping Chu, Xiang-ming Zha

**Affiliations:** 1 Department of Cell Biology and Neuroscience, University of South Alabama College of Medicine, Mobile, Alabama, United States of America; 2 State Key Lab of New Drug & Pharmaceutical Process, Shanghai Institute of Pharmaceutical Industry, Shanghai, China; 3 Department of Urology, The Third Hospital of Hebei Medical University, ShiJiaZhuang, HeBei Province, China; 4 Departments of Basic Medical Sciences and Anesthesiology, University of Missouri- Kansas City School of Medicine, Kansas City, Missouri, United States of America; 5 Department of Mathematics and Statistics, University of South Alabama, Mobile, Alabama, United States of America; University of Houston, United States of America

## Abstract

Acid-sensing ion channel-1a (ASIC1a), the primary proton receptor in the brain, contributes to multiple diseases including stroke, epilepsy and multiple sclerosis. Thus, a better understanding of its biogenesis will provide important insights into the regulation of ASIC1a in diseases. Interestingly, ASIC1a contains a large, yet well organized ectodomain, which suggests the hypothesis that correct formation of domain-domain interactions at the extracellular side is a key regulatory step for ASIC1a maturation and trafficking. We tested this hypothesis here by focusing on the interaction between the first transmembrane domain (TM1) and the thumb of ASIC1a, an interaction known to be critical in channel gating. We mutated Tyr71 and Trp287, two key residues involved in the TM1-thumb interaction in mouse ASIC1a, and found that both Y71G and W287G decreased synaptic targeting and surface expression of ASIC1a. These defects were likely due to altered folding; both mutants showed increased resistance to tryptic cleavage, suggesting a change in conformation. Moreover, both mutants lacked the maturation of *N*-linked glycans through mid to late Golgi. These data suggest that disrupting the interaction between TM1 and thumb alters ASIC1a folding, impedes its glycosylation and reduces its trafficking. Moreover, reducing the culture temperature, an approach commonly used to facilitate protein folding, increased ASIC1a glycosylation, surface expression, current density and slowed the rate of desensitization. These results suggest that correct folding of extracellular ectodomain plays a critical role in ASIC1a biogenesis and function.

## Introduction

Acid-sensing ion channels (ASICs) are a family of proton-gated cation channels that conduct primarily Na^+^ [1], [2]. Among all 6 ASICs (1a, 1b, 2a, 2b, 3, and 4) identified, ASIC1a [3] plays a particularly important role in determining H^+^-activated responses in the brain. Deleting the *ASIC1a* gene renders CNS neurons unresponsive to acidic pHs as low as pH 5 [4]. Functionally, ASIC1a contributes to synaptic plasticity, learning and fear [5], [6], [7], [8], [9]. More importantly, recent studies show that ASIC1a plays a critical role in acidosis-induced neuronal damage and contributes to several neurological diseases including brain ischemia, epilepsy, and multiple sclerosis [10], [11], [12], [13], [14], [15], [16]. A better understanding of ASIC1a regulation will likely provide important new insights into its role in neuron physiology and pathophysiology.

The biogenesis process in general plays an important role in regulating channel trafficking and function. Common mechanisms to regulate protein biogenesis include intracellular interactions with accessory proteins. Interestingly, the extracellular portion of ASIC1 contains a complex, yet well organized, ectodomain [17], [18], suggesting that folding of the extracellular domain is a critical step for ASIC biogenesis. Since the correct three-dimensional (3D) conformation of the receptor protein depends heavily upon interactions between different structural domains, we hypothesize that domain-domain interactions within the ectodomain of ASIC1a is important for its glycosylation and trafficking. For the present study, we focused on the interaction between TM1 and the thumb region of ASIC1a as it is known that hydrophobic interactions between these two regions is critical for ASIC1a gating [19], [20], [21]. However, it remains unclear whether the same interactions affect ASIC1a biogenesis.

We mutated Trp287 and Tyr71, two key residues regulating the TM1-thumb interaction in mouse ASIC1a [19], and found that the mutations changed ASIC1a folding, decreased ASIC1a glycosylation, and reduced synaptic targeting and surface expression of ASIC1a. Conversely, lowering the culture temperature, a common intervention to facilitate folding, increased glycosylation, surface expression and current amplitude of ASIC1a. These results demonstrate that hydrophobic interactions within the ectodomain of ASIC1a are critical for glycosylation and trafficking of ASIC1a and further suggest that folding is a rate-limiting step in ASIC1a biogenesis.

## Results

### Mutating Trp287 or Tyr71 reduces dendritic targeting and surface expression of ASIC1a

**Figure 1:**
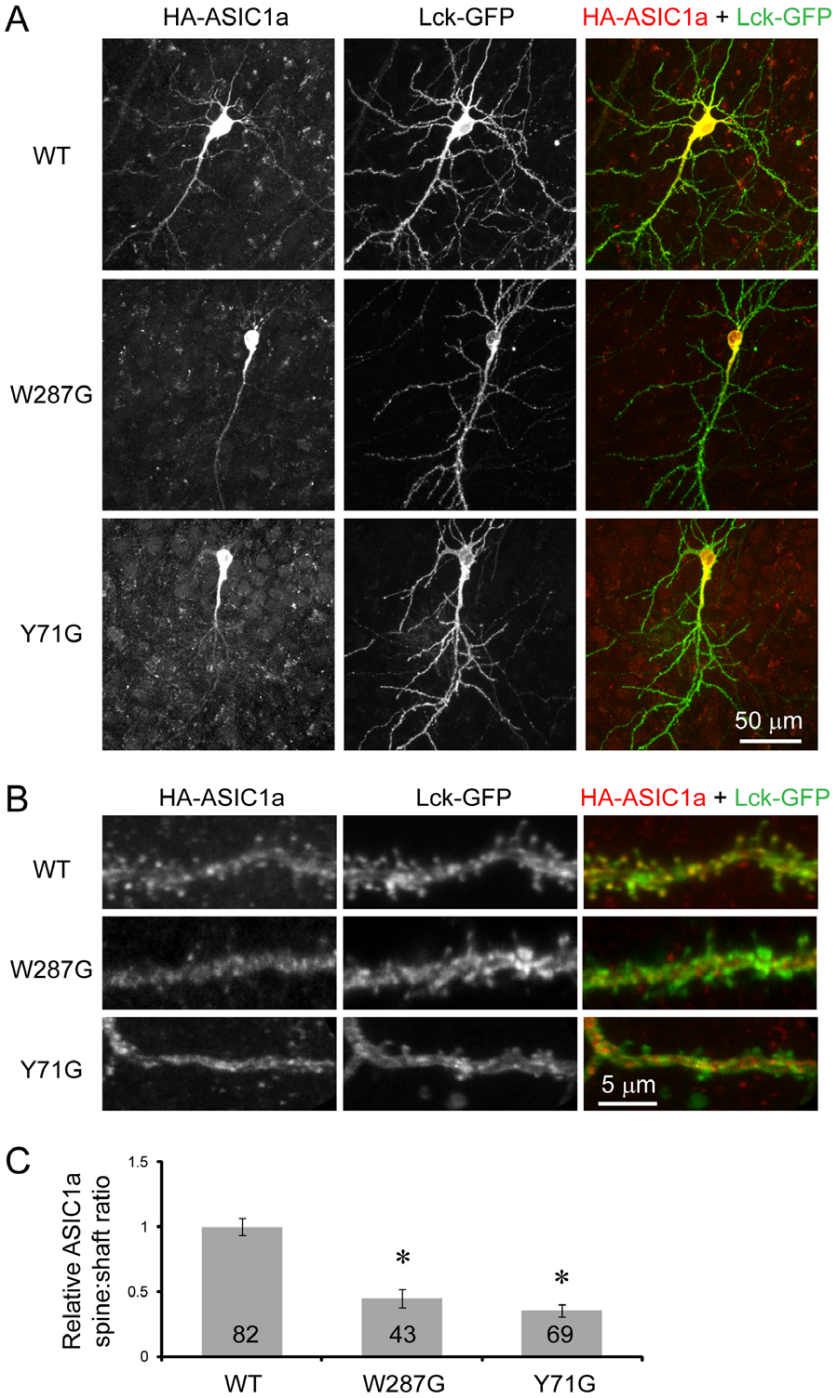
Mutating Tyr71 and Trp287 reduces dendritic targeting of ASIC1a. *ASIC2-/-* hippocampal slices were co-transfected with HA-tagged wild-type ASIC1a, W287G or Y71G mutants together with a membrane targeted Lck-GFP [22]. Localization of transfected ASIC1a was visualized by immunofluorescence using an anti-HA antibody. (**A**) Confocal images showing the overall distribution of ASIC1a in transfected neurons. Left panel shows ASIC1a localization, middle panel shows GFP fluorescence, right panel shows the merged image (HA immunofluorescence in red and GFP fluorescence in green). Note the presence of wild-type ASIC1a in most dendritic branches, while mutant ASIC1a localized primarily in the cell body and proximal dendrites. (**B**) High magnification view of a segment of apical dendrite. Note the presence of wild-type but not mutant ASIC1a in most dendritic spines. (**C**) Quantification of relative ASIC1a spine:shaft ratio. The ratio of ASIC1a spine:shaft was normalized to that of the membrane-targeted Lck-GFP. The normalized ratio of wild-type ASIC1a was set arbitrarily to 1. Numbers on the bar indicate the total number of spines quantified from 5 wild-type, 4 71G, and 5 287G transfected neurons from two separate experiments. Asterisks indicate significant differences (P<0.0001, unbalanced ANOVA).

To answer whether the interaction between TM1 and the thumb affects ASIC1a trafficking, we generated W287G and Y71G mutants of mouse ASIC1a. Similar mutants have been shown previously to disrupt the interaction between TM1 and the thumb [19]. We first studied the localization of W287G and Y71G in organotypic hippocampal slices. We transfected *ASIC2-/-* slices with HA-tagged wild-type or mutant ASIC1a together with Lck-GFP [22], a membrane-targeted GFP which facilitates the visualization of transfected neurons. We used *ASIC2-/-* slices because our previous studies have shown that endogenous ASIC2 regulates ASIC1a targeting in slice neurons [23]. Thus, using *ASIC2-/-* slices can ensure that the effect we observe is due to changes in ASIC1a subunit, as opposed to a changed association with ASIC2 subunits. It will also alleviate the concern that endogenous ASIC2 may mask any potential trafficking defects that the mutants have. Similar to earlier studies, wild-type ASIC1a localized to most dendrites and spines (Fig. 1A,B). In contrast, both W287G and Y71G exhibited diminished expression in distal dendrites and in most dendritic spines. To analyze quantitatively the enrichment of ASIC1a in spines, we quantified the relative fluorescence intensity of wild-type and mutant ASIC1a. W287G and Y71G had significantly reduced levels in spines (Fig 1B,C). These data suggest that the interaction between Trp287 and Tyr71 is necessary for targeting of ASIC1a to dendrites and spines in neurons.

**Figure 2:**
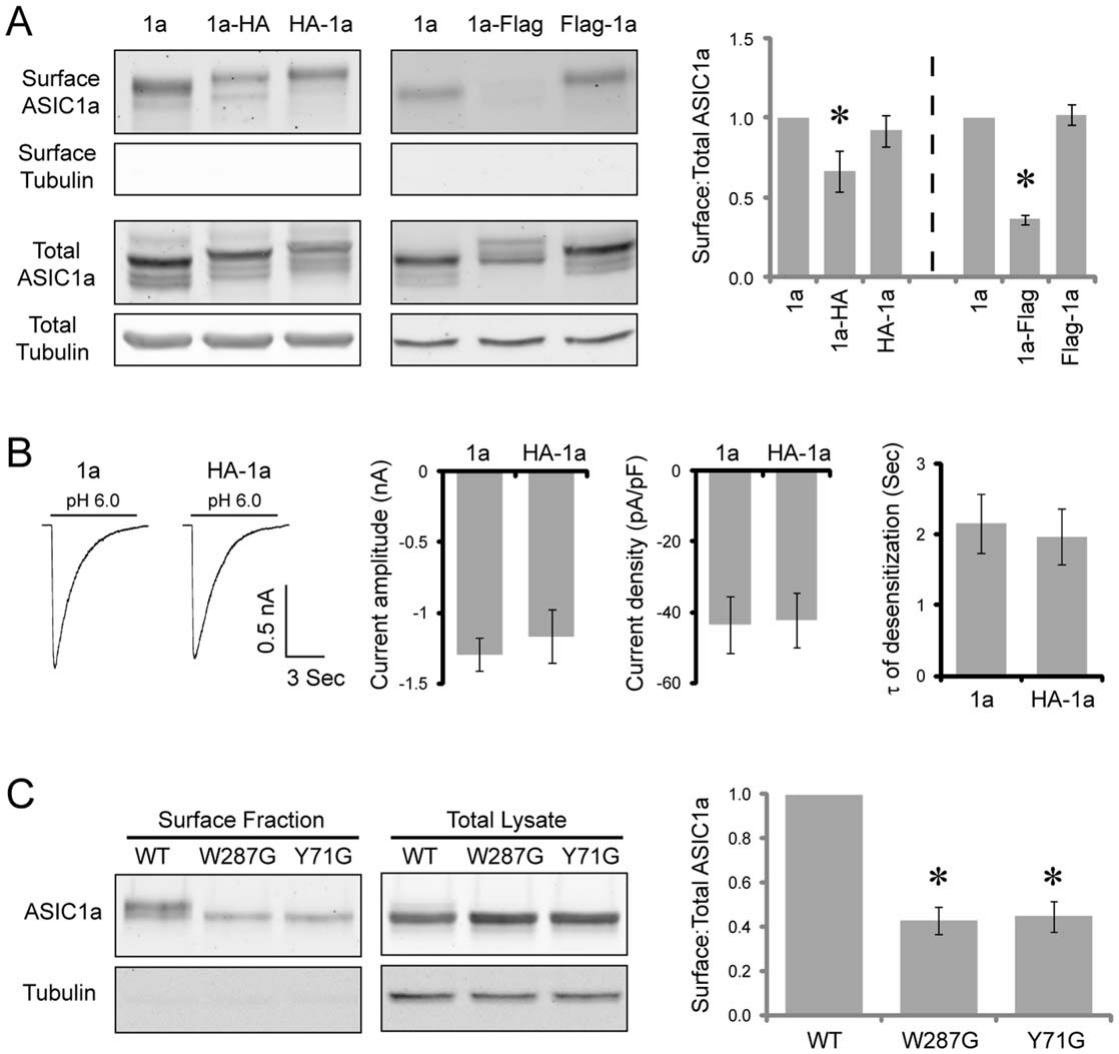
Mutating Tyr71 and Trp287 reduces ASIC1a surface expression. (**A**) The effect of N- and C-terminal tags on surface expression of ASIC1a. CHO cells were transfected with untagged, N-terminal or C-terminal HA- or Flag-tagged ASIC1a as indicated. Surface and total proteins were blotted with a goat anti-ASIC1 and a mouse anti-tubulin antibody, as described in the Methods. Right panel shows the quantification of the surface:total ratio of untagged and tagged ASIC1a. The dotted line indicates that the controls for HA and Flag tagged experiments were different. Average surface:total ratio for WT was 4.4%±0.5%. For easy comparison, the ratio of WT was set arbitrarily to 1. (**B**) Typical traces and quantification of pH6-activated current recorded from cells expressing untagged (n = 6) or N-terminal HA-tagged (n = 7) ASIC1a. (**C**) Western blot and quantification showing surface:total ratio of HA-tagged wild-type ASIC1a, W287G, or Y71G mutants. Asterisks indicate a significant difference from controls (P<0.01, unbalanced ANOVA).

Besides subcellular targeting, surface expression is another key aspect of ion channel trafficking. However, it is technically challenging to achieve high transfection efficiency in neurons, making it difficult to perform quantitative analysis of the surface levels of wild-type and mutant ASIC1a. We therefore switched to Chinese hamster ovary (CHO) cells for surface biotinylation studies. One technical note is that previous studies have used both N- and C-terminal tagged ASICs to study ASIC surface expression [20], [24], [25]. However, it is not known whether these tags differentially affect ASIC surface expression. Thus, we quantified the expression and surface levels of N- and C-terminal tagged ASIC1a. We transfected CHO cells with untagged or N- or C-terminal-tagged ASIC1a, performed surface biotinylation, and precipitated surface proteins with NeutrAvidin beads. 4.4%±0.5% of untagged ASIC1a reached the cell surface. Tagging the N-terminus had no effect on ASIC1a expression or surface levels (Fig. 2A). In contrast, tagging the C-terminus significantly reduced surface:total ratio of ASIC1a. We further studied acid-activated current and found that N-terminal HA tag did not change the amplitude, density or desensitization rate of pH 6-induced ASIC current (Fig. 2B). Moreover, our previous results demonstrate that N-terminal tagged ASICs show a localization pattern similar to that of endogenous proteins [23], [26]. Together, these data argue that adding N-terminal small epitopes are better suited for studying ASIC trafficking. Therefore, we used N-terminal tagged ASIC1a for the remaining experiments.

**Figure 3:**
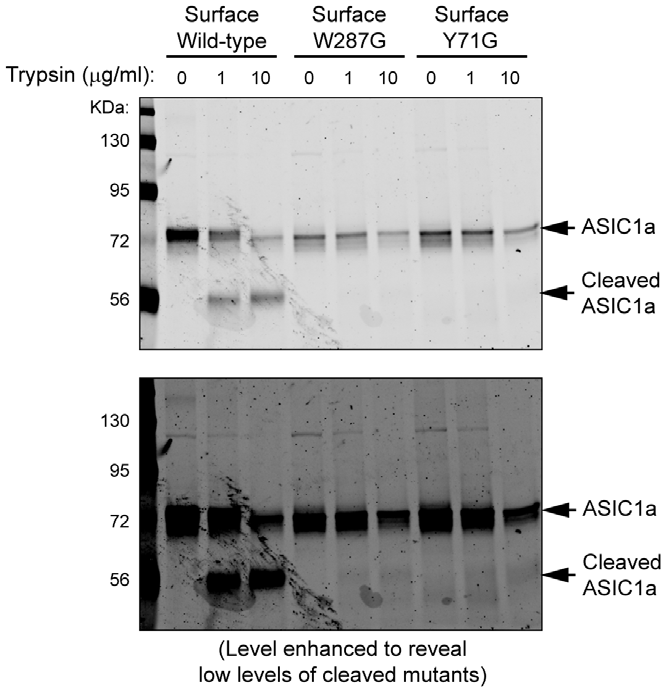
W287G and Y71G show increased resistance to tryptic cleavage. CHO cells were transfected with wild-type, W287G or Y71G ASIC1a. Following biotinylation of surface proteins, cells were treated with 0, 1, or 10 µg/ml trypsin for 20 mins. Surface proteins were isolated and blotted for ASIC1a. Note that wild-type ASIC1a at the surface was cleaved by trypsin in a dosage-dependent manner. Also note that the amount of uncleaved mutants was comparable to that of uncleaved WT, although the mutants only had a small fraction of cleaved population (see the bottom image with level enhanced). The decrease in loading in trypsin treated lanes was likely due to the loss of some cells during the treatment. Blots shown are representative from three separate experiments.

**Figure 4:**
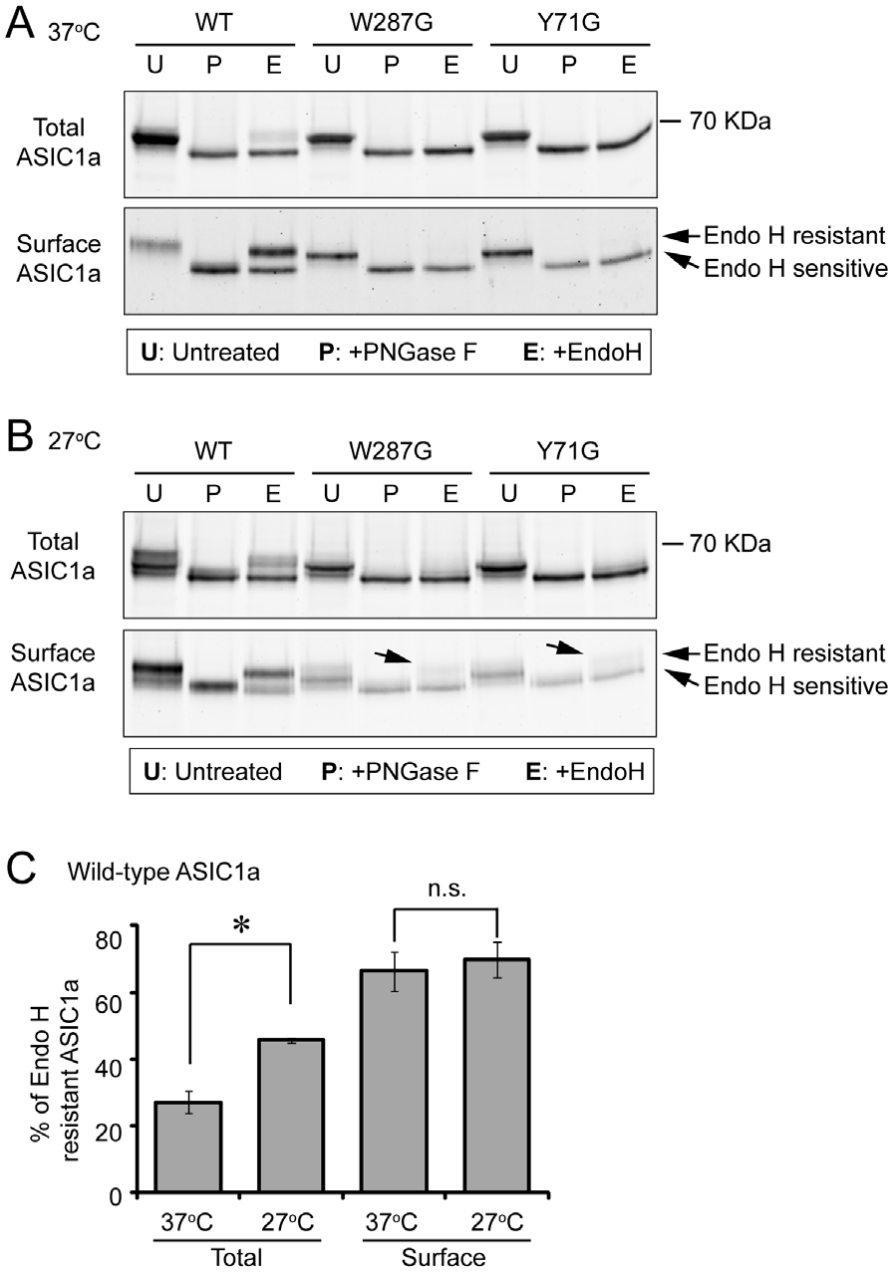
W287G and Y71G reduces ASIC1a glycosylation while a reduced culture temperature has an opposite effect. (**A & B**) Representative blots showing the effect of a reduced culture temperature on ASIC1a glycosylation. CHO cells were transfected with wild-type ASIC1a, W287G or Y71G mutant. Surface biotinylation was performed after culturing at 37°C (A) or 27°C (B) for 24 hrs. Lysates were treated with PNGase F or Endo H as indicated, followed by NeutrAvidin precipitation. Surface and total fraction were blotted for ASIC1a. The relative position of Endo H-resistant and -sensitive ASIC1a was indicated by arrows. Note that 27°C increased the percentage of Endo H-resistant wild-type ASIC1a in total lysate. Also note the appearance of Endo H-resistant W287G and Y71G at the cell surface at 27°C (arrows). (**C**) Quantification of Endo H-resistant wild-type ASIC1a at 37°C and 27°C. Asterisk indicates significant difference (P = 0.025); n.s. stands for not significant.

We transfected CHO cells with wild-type ASIC1a, W287G or Y71G mutants and analyzed the surface and total fraction of the protein. We noticed that there was a small shift in the apparent molecular weight between surface WT and mutant ASIC1a (also see Figures 3 & 4). We speculate that this difference may be due to a reduced glycosylation of the mutants (see Fig. 4). Both W287G and Y71G had significantly reduced surface:total ratio (Fig. 2C). In summary, our data in hippocampal slices and CHO cells showed that mutating Tyr71 or Trp287 reduced both synaptic targeting and surface expression of ASIC1a.

### W287G and Y71G mutants show altered ASIC1a folding and reduced N-linked glycosylation

If our overall hypothesis is correct, we expect that W287G and Y71G will disrupt ASIC1a folding and reduce its glycosylation. To answer if this is the case, we first asked whether W287G and Y71G show a change in 3D conformation, which indicates a change in folding. We did surface biotinylation, performed limited tryptic cleavage of surface proteins, then lysed the cells and analyzed surface proteins with Western blot. The working hypothesis is that a change in channel conformation will change the sensitivity of these channels to proteases [27]. A similar method has been used to probe conformational changes of other ion channels [28]. Twenty minute treatment with trypsin cleaved wild-type ASIC1a in a dose-dependent manner (Fig. 3). Consistent with an earlier report [29], the major product of tryptic cleavage was a C-terminal fragment of ∼55KDa. After the treatment with 10 µg/ml trypsin, the ratio of cleaved:uncleaved WT ASIC1a was 2.22±0.29∶1. In contrast, although we could detect low levels of cleaved W287G and Y71G on the gel, both showed dramatically reduced sensitivity to trypsin; the ratio of cleaved:uncleaved W287G and Y71G was 0.07±0.01∶1 and 0.06±0.02∶1, respectively, both mutants significantly (p<0.02, paired t-test, n = 3) lower than that of wild-type. This increased resistance to proteolysis is consistent with a changed 3D conformation of the mutants and suggests that disrupting the TM1-thumb interaction leads to an impaired folding.

A defective folding generally increases ER retention and slows protein maturation. To verify this prediction, we treated cell lysates with Endo H and PNGase F. Endo H removes immature core-glycosylated *N*-linked glycans. In contrast, PNGase F removes all *N*-linked glycans on proteins [30], [31]. As expected, PNGase F treatment converted both wild-type and mutant ASIC1a into the same fastest migrating species, demonstrating that the difference in migration patterns between WT and mutants was due to differences in glycosylation (Fig. 4A). For wild-type ASIC1a, 66% and 24% of surface and total ASIC1a, respectively, were resistant to Endo H treatment (Fig. 4C). In contrast, Endo H converted all W287G and Y71G into the fastest migrating species (Fig. 4A), indicating that these ASIC1a mutants contained primarily core-glycans.

### Temperature regulates glycosylation and surface expression of ASIC1a

Our data suggest that ASIC1a has an inefficient glycosylation and trafficking, which was further reduced by mutations that disrupt ASIC1a folding. If true, interventions that facilitate folding should increase ASIC1a biogenesis. One well established method to enhance protein folding is lowering the culture temperature. For example, the ΔF508 of cystic fibrosis transmembrane conductance regulator (CFTR), a folding mutant which also shows defects in glycosylation and surface expression, is partially rescued by reducing the culture temperature [32]. Therefore, we hypothesized that a reduced temperature would increase ASIC1a glycosylation and rescue the trafficking defects seen in W287G and Y71G. To test this hypothesis, we transfected CHO cells with wild-type ASIC1a and cultured the cells for 24 hrs at 37°C or 27°C. Culturing at 27°C increased the amount of fully glycosylated ASIC1a; 46% of the wild-type ASIC1a was resistant to Endo H treatment, as opposed to 24% at 37°C (Fig. 4A–C). Interestingly, the % of Endo H-resistant ASIC1a at the cell surface remained the same after 24 hrs at 27°C (Fig. 4C). In contrast to wild-type ASIC1a, at 27°C, the mutants in the total fraction did not show obvious induction of Endo H-resistant populations. However, at 27°C, a small fraction of the mutants at the cell surface exhibited resistance to Endo H (Fig. 4B). This result indicates that 27°C, though less efficiently, induced fully glycosylated forms of mutants, which were preferentially trafficked to cell surface.

**Figure 5:**
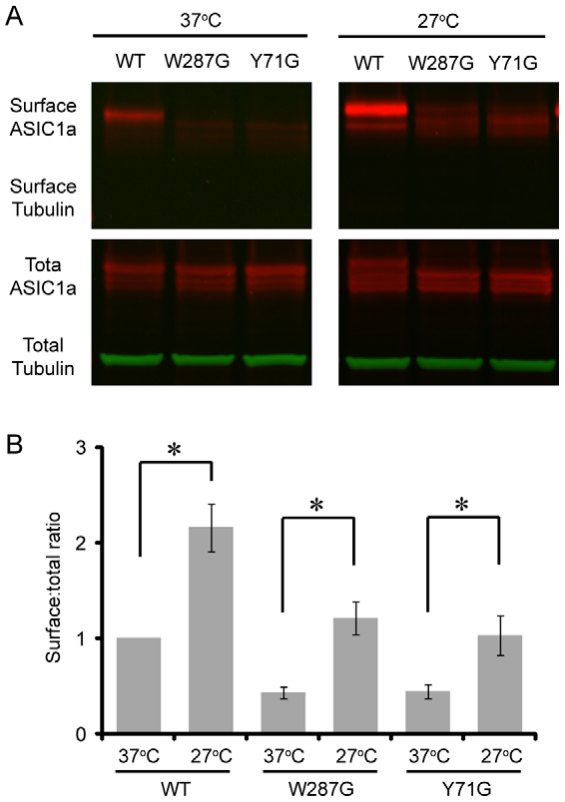
Lowering the culture temperature increases ASIC1a surface expression. Representative Western blot (**A**) and quantification (**B**) showing the effect of 24 hrs of 27°C on surface expression of wild-type and mutant ASIC1a. ASIC1a (red channel) and tubulin (green channel) were blotted and detected on the same blot as described in Methods. Surface and total ASIC1a were analyzed as described in the previous figures. Asterisks indicate significant differences from 37°C (P<0.05, student's t-test, n = 5−6).

These results suggest that reducing the culture temperature facilitates the folding and subsequent maturation of ASIC1a through the secretory pathway, and further predicts an increase in surface expression of both wild-type and mutant ASIC1a. Therefore, we studied ASIC1a surface expression after 24 hrs incubation at 37°C or 27°C. As expected, reducing the culture temperature to 27°C significantly increased the surface:total ratio of both wild-type and mutant ASIC1a (Fig. 5A, B).

### Lowering culture temperature increases the current amplitude and slows the desensitization rate of ASIC1a

**Figure 6:**
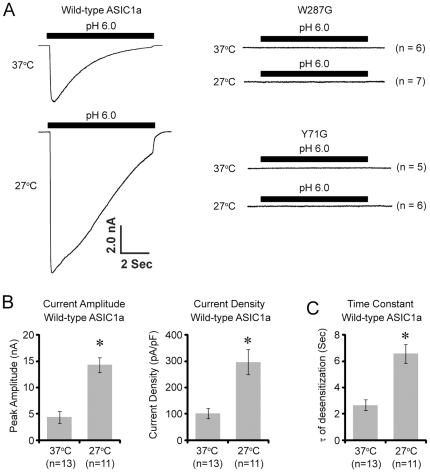
Lowering the culture temperature increases the current amplitude and slows the rate of desensitization of ASIC1a. CHO cells were co-transfected with wild-type, W287G or Y71G ASIC1a together with GFP. Cells were cultured overnight at 37°C followed by 24 hrs at 37°C or 27°C. All recordings were performed at the same temperature (∼23°C). (**A**) Representative pH 6-induced current from cells expressing wild-type or mutant ASIC1a. (**B & C**) Quantification of the effect of 27°C on current amplitude (B) and the rate of desensitization (C) of wild-type ASIC1a. Asterisks indicate significant differences from 37°C (P<0.01, student's t-test).

The increase in surface levels of wild-type ASIC1a at 27°C predicts an increase in current amplitude. While previous studies show that W287G and Y71G are proton-insensitive, the appearance of the fully glycosylated form of W287G and Y71G at the cell surface raised the question of whether these mutants become functional after culturing at 27°C [19], [20]. To address these questions, we studied acid-activated currents of wild-type and mutant ASIC1a after 24 hrs of culturing at 27°C. Since an acute temperature change itself affects ASIC1a current [33], [34], [35], we studied acid-activated currents at the same temperature (∼23°C), regardless of the temperature at which the cells were initially cultured. Culturing at 27°C significantly increased the current amplitude and slowed the rate of desensitization of wild-type ASIC1a (Fig. 6A–C). Similar to earlier reports [19], [20], pH 6 application failed to elicit any current from W287G or Y71G expressing cells, even after 24 hrs culturing at 27°C (Fig. 6A). These results show that a reduced temperature failed to rescue the gating deficiency of these mutants, but had a profound effect on the function of wild-type ASIC1a.

## Discussion

Our results indicate that one critical step for ASIC1a biogenesis is the formation of correct domain-domain interactions in the ectodomain. Lowering the culture temperature, a common method to facilitate folding, increased ASIC1a glycosylation, surface expression, and current density. Conversely, disrupting a key hydrophobic interaction between TM1 and the thumb led to a change in ASIC1a folding, reduced ASIC1a glycosylation, impaired ASIC1a trafficking and abolished channel function. These data suggest that one rate-limiting step for ASIC1a biogenesis is its folding and maturation, which are tightly associated with the formation of hydrophobic interactions within its ectodomain.

With a combination of computational analysis and electrophysiological approaches, Yang et al. demonstrated that the Tyr at the end of TM1 and Trp at the bottom of the thumb show correlative movement in response to protons [19]. This interaction apparently plays a critical role in ASIC1a gating; mutating these two residues renders ASIC1a proton insensitive [19], [20]. Our data here provide additional support for above modeling; the tryptic cleavage data suggest that W287G and Y71G achieve a conformation different from the wild-type channels. While these results emphasize the importance of the thumb-TM1 interaction in channel conformation, our biochemical and immunofluorescence analysis revealed that the same interaction is also important for ASIC1a glycosylation, surface expression and dendritic targeting. These results suggest that mutations affecting ASIC1a gating interfere with channel maturation and trafficking. This is an important point which needs to be considered when designing functional studies using "dead" ASIC channels.

In a previous study, mutating the glycosylation sites on ASIC1a reduces its current amplitude [36]. Interestingly, reducing the culture temperature here increased ASIC1a glycosylation, surface expression and acid-activated current density. In contrast, both the W287G and Y71G mutants reduced ASIC1a glycosylation and disrupted ASIC1a channel function. Taken together, these data suggest that intervening the glycosylation process is one efficient way to regulate ASIC1a function. Further investigation of the mechanisms controlling this process will be important for understanding its role in diseases. Of note, diseases that induce ER stress generally regulate the expression of chaperone proteins and have profound effects on protein folding and maturation [37]. Several heat shock proteins regulate ASIC2 and ENaC trafficking [38], [39], [40]. One recent study further showed that serum starvation, a common stress stimulus *in vitro*, leads to a rapid increase in ASIC1a surface levels [24]. These results suggest a tight relationship between ER stress, the level of certain ER resident molecular chaperones and the biogenesis of ASIC1a.

In parallel to an increase in surface expression, we observed a change in current amplitude and property at 27°C. Interestingly, several earlier studies also reported that a reduced temperature increases ASIC current amplitude and slows its kinetics [33], [34], [35]. However, the phenomenon we observed here is different from the previous reports. In previous studies, cells were cultured at a constant temperature, but the current was studied at different temperatures in the recording chamber. The changes in current in those studies were acute, probably due to acute changes in channel conformation. Here, we cultured cells for 24 hrs at two different temperatures (27°C or 37°C) but recorded their current at the same temperature (∼23°C). Our current observations thus reflect a novel phenomenon, distinct from the acute effects reported earlier [33], [34], [35]. The effects we observed most likely reflect a change in covalent modifications, e.g., differential glycosylation, in ASIC1a during its biogenesis. Of note, we cannot rule out the possibility that increased surface expression at 27°C contributed to the slowed desensitization. The detailed mechanism for this phenomenon, however, is beyond the scope of this paper.

Our results suggest that disrupting ASIC1a folding reduces its surface expression and impairs its localization to dendrite and dendritic spines, the sites for the majority of excitatory neurotransmission in the brain. These changes correlate with a decrease in glycosylation, suggesting that *N*-glycosylation is important for ASIC1a trafficking and function. Further, these results suggest that factors regulating protein folding play a critical role in determining the migration of ASIC1a through the secretory pathway, and thus have important impact on the functional outcome of acidosis.

## Materials and Methods

### Mice

*ASIC2-/-* mice on a congenic C57/BL6 background were kindly provided by Dr. Michael Welsh [41]. Wild-type and ASIC2 knockout mice were maintained as described earlier [23]. Animal care met National Institutes of Health standards and all procedures were approved by the University of South Alabama Animal Care and Use Committee (approved animal protocols #09032 and 09033).

### Constructs and Reagents

Lck-GFP was kindly provided by Dr. Steven Green. N-terminal HA- and Flag-tagged wild-type mouse ASIC1a has been described earlier [25], [26]. C-terminal tagged ASIC1a constructs (ASIC1a-HA and ASIC1a-Flag) were generated by PCR-mediated subcloning. HA-tagged W287G and Y71G mutants were generated with a Quickchange mutagenesis kit (Stratagene, La Jolla, CA), following manufacturer's instructions. All constructs were verified by sequencing. Rabbit anti-ASIC1 was kindly provided by Dr. John Wemmie [42]. Other antibodies used were: rat monoclonal anti-HA (Roche, Switzerland), mouse monoclonal anti-HA (Santa Cruz, Santa Cruz, CA and Syd Labs, Malden, MA), mouse monoclonal anti-Tubulin (University of Iowa Developmental Hybridoma Bank), goat anti-ASIC1 (Santa Cruz), Alexa 488-, 568-, 680-, and 800-conjugated secondary antibodies (Invitrogen, CA & Li-cor, Lincoln, NE), and Dylight 488-, 680- and 800- conjugated secondary antibodies (Pierce, Rockford, IL). Other reagents used: Endo H and PNGase F (New England Biolabs, Ipswich, MA); Trypsin (Sigma); NHS-sulfo-biotin, NHS-sulfo-LC-biotin and NeutrAvidin Beads (Pierce); culture media and serum (HyClone or Invitrogen); and lipofectamine 2000 (Invitrogen).

### Hippocampal slice culture, transfection and immunofluorescence

Organotypic hippocampal slice culture, biolistic-mediated transfection and immunofluorescence was done similar to what was described earlier [26], [43], [44]. Briefly, hippocampi for postnatal day 5–7 pups were cut into 350 µm thick sections and cultured in Falcon polyethylene terephthalate-etched membrane culture inserts containing 1 µm pores (Fisher), at a density of 5–6 slices per insert. Slices were maintained in filter culture medium (FCM) (25% horse serum, 25% Hanks Balanced Salt Solution, 50% MEM, supplemented with 2 mM glutamax, 1.5 mg/ml glucose, 44 mg/ml NaHCO_3_ and 10 U/ml penicillin-streptomycin). Slices were grown in a 5% CO_2_ humidified incubator. The medium was changed every 2–3 days. Transfection was performed after 8–10 days in culture with a Helios genegun (Bio-Rad) at 70–75 psi, similar to what was described earlier [23], [26]. All slices were fixed two days after transfection, at an age equivalent of 15–16 days age (e.g., P6 + 9–10 days *in vitro* (DIV)).

### CHO cell culture and transfection

CHO-K1 cells were purchased from ATCC and used between passages 3 to 14. CHO cells were grown in F-12K supplemented with 10% fetal bovine serum in a humidified 5% CO_2_ incubator. For biochemistry experiments, cells were plated onto 60 mm dishes at a density of 3−4×10^4^ cells/cm^2^ (6−8×10^5^ per 60 mm dish) and transfected using Lipofectamine 2000, following the manufacturer's instruction. For electrophysiology, cells were grown in 35 mm dishes at a density of 50 to 70% and transfected using Fugene HD following the manufacturer's instruction.

### Surface Biotinylation, De-glycosylation, NeutrAvidin Pull-down and Western Blot

Surface biotinylation and NeutrAvidin pulldown was performed similar to what was described earlier [25]. Briefly, cells were washed 3 times with ice-cold PBS+/+, followed by 30 min incubation at 4°C in 1.5 ml of PBS+/+ containing 0.5 mg/ml Sulfo-NHS-LC-biotin. Cells were washed once with cold PBS+/+ and the reaction was quenched by 100 mM glycine in PBS+/+. Cells were lysed in 300 µl NeutrAvidin lysis buffer (PBS, 1% Triton, 0.5% SDS, 0.5 mg/ml NEM, with protease inhibitors). Cell lysates were sonicated briefly and centrifuged at full speed with a desktop centrifuge for 10 min at 4°C. For precipitation of surface proteins, 40 µl of NeutrAvidin agarose beads were added to 200 µl of cell lysate (approximately 600 µg of proteins) and the precipitation was carried out overnight at 4°C with gentle rotation, followed by 3 washes with PBS containing 1% Triton. Surface fraction was eluted with 80 µL of 2× sample buffer containing 5% β-mercaptoethanol. Total lysate was mixed with an equal volume of 3× sample buffer. Equal volume of surface and total fraction was loaded per lane so the loading into the surface fraction is about 5 times that of total.

De-glycosylation with Endo H or PNGase F was performed following the manufacturer's instructions. The samples were denatured in 1X denaturing buffer at 95°C for 5–10 min and cooled to room temperature. For PNGase F digestion, NP-40 was added to a final concentration of 1%. The reaction mixture (200 µl lysate, approximately 600 µg protein) was incubated overnight at 37°C with 3 µl of PNGase F ( = 22 IUB milli units) or Endo H ( = 150 IUB milli units). Following PNGase F or Endo H treatment, isolation and analysis of surface proteins were performed as described above.

The samples were separated by 8% or 10% SDS-PAGE and transferred to nitrocellulose membranes. Blotting was performed according to instructions of the Odyssey Imaging System (Li-cor), similar to what was described earlier [25]. Antibody dilutions were: rabbit anti-ASIC1a 1∶5K-30K; goat anti-ASIC1 1∶500; monoclonal anti-HA 1∶1K–2K; monoclonal anti-tubulin, 1∶30,000; goat anti-rabbit Alexa 680 1∶16,000 and goat anti-mouse Dylight 800 1∶16,000. Blots were imaged using an Odyssey Infrared Imaging System according to manufacturer's instructions. Densitometry of imaged bands was performed as described earlier [23], [26].

### Limited Tryptic Cleavage of Surface Proteins

Following surface biotinylation, CHO cells were incubated in PBS+/+ containing 0, 1, or 10 µg/ml of trypsin for 20 min at room temperature. Cells were washed 3 times with PBS+/+. Cells were lysed and surface proteins were precipitated and analyzed as described above.

### Confocal microscopy and quantification of immunofluorescence images

Confocal images were captured using a laser scanning microscope (Nikon A1), similar to procedures described earlier [43]. Illumination was provided by an argon (Ar, 458, 488, 514 nm lines) and a 561 diode laser. For ASIC1a immunofluorescence, green and red channels were imaged sequentially to eliminate bleed-through. Alexa 488 and GFP were imaged with 488 nm excitation and a 525/50 emission filter. Alexa 568 was imaged with 561 nm excitation and a 595/50 emission filter. Images were captured with a 20x/0.75 multi-immersion lens or a 63x/1.2 PL APO water lens. To visualize spines, a series of high-resolution images (1024×256 to 1024×1024 pixel array) were captured at a z-step of 0.4–0.5 µm with an additional electronic zoom of 4. Each captured image was an average of 4 scans in a single plane. For each transfected neuron, one middle segment of an apical dendrite (about 100–200 µm away from the cell body layer) was imaged and used for spine analysis. The image field of view is approximately 50–70 µm and thus covers a large fraction of the medial portion of an apical dendrite. Raw images were exported and further analyzed in ImageJ.

### Electrophysiology

Whole-cell patch-clamp recordings were performed as described previously [45], [46], [47], [48], [49]. Briefly, patch electrodes were constructed from thin-walled borosilicated glass (1.5 mm diameter, WPI, Sarasota, FL) on a two-stage puller (PC-10, Narishige, Tokyo, Japan). The resistance of the patch electrode ranged from 3 to 6 MΩ when filled with intracellular solution. Whole-cell currents were elicited by a drop in pH from 7.4 to 6.0 at a holding potential of −60 mV and recorded using Axopatch 200B amplifiers (Axon CNS, Molecular Devices, Foster City, CA). Data were filtered at 2 kHz and digitized at 5 Hz using Digidata 1440 DAC units (Axon CNS, Molecular Devices, Foster City, CA). The on-line acquisition was done using pCLAMP software (Version 10.2, Axon CNS, Molecular Devices, Foster City, CA). Standard extracellular fluid contained (in mM): 140 NaCl, 5.4 KCl, 2.0 CaCl_2_, 1.0 MgCl_2_, 20 HEPES, and 10 glucose (pH 7.4; 320 ∼330 mOsm). For solutions with a pH≤6.0, MES was used instead of HEPES for more reliable pH buffering. The pipette solution contained (mM) 140 K Gluconate, 10 HEPES, 11 EGTA, 2 TEA, 1 CaCl_2_, 2 MgCl_2_, and 4 K_2_ATP (pH 7.2∼7.3; 290∼300 mOsm). A multi-barrel perfusion system (SF-77, Warner Instrument Co., CT) was employed to achieve a rapid exchange of extracellular solutions. During each experiment, a voltage step of –10 mV from the holding potential was applied periodically to monitor the cell capacitance and the access resistance. Recordings in which either the access resistance or the capacitance changed by more than 10% during the experiment were excluded from data analysis. To determine the time constant of the desensitizing portion of the ASIC currents, pH 6.0-activated currents were fitted by a single, standard exponential equation using Clampfit 10.2.

### Statistical analysis

For paired comparisons, we used two-tailed Student's t-test. For multiple comparisons, we used SAS procedure GLM to perform ANOVA for unbalanced data and perform custom hypotheses tested with CONTRAST statement. Data were reported as mean±s.e.m. for the number of samples indicated.
